# Phosphorylated STAT5 regulates p53 expression via BRCA1/BARD1-NPM1 and MDM2

**DOI:** 10.1038/cddis.2016.430

**Published:** 2016-12-22

**Authors:** Zhuo Ren, Joeri L Aerts, Hugo Vandenplas, Jiance A Wang, Olena Gorbenko, Jack P Chen, Philippe Giron, Carlo Heirman, Cleo Goyvaerts, Eldad Zacksenhaus, Mark D Minden, Vuk Stambolic, Karine Breckpot, Jacques De Grève

**Affiliations:** 1 Laboratory of Medical and Molecular Oncology (LMMO), Department of Medical Oncology, Vrije Universiteit Brussel, Brussels, Belgium; 2 Department of General Surgery, The First People's Hospital of Shanghai, Shanghai Jiaotong University, Shanghai, China; 3 Department of Medical Oncology, Oncologisch Centrum of the Universitair Ziekenhuis Brussel, Vrije Universiteit Brussel, Brussels, Belgium; 4 Princess Margaret Cancer Centre, University Health Network, Toronto, ON, Canada; 5 Laboratory of Molecular and Cellular Therapy, Department of Physiology and Immunology, Vrije Universiteit Brussel, Brussels, Belgium; 6 Department of Medicine and Medical Biophysics, University of Toronto, Toronto, ON, Canada; 7 Toronto General Research Institute, University Health Network, Toronto, ON, Canada

## Abstract

Signal transducer and activator of transcription 5 (STAT5) and nucleophosmin (NPM1) are critical regulators of multiple biological and pathological processes. Although a reciprocal regulatory relationship was established between STAT5A and a NPM–ALK fusion protein in T-cell lymphoma, no direct connection between STAT5 and wild-type NPM1 has been documented. Here we demonstrate a mutually regulatory relationship between STAT5 and NPM1. Induction of STAT5 phosphorylation at Y694 (P-STAT5) diminished NPM1 expression, whereas inhibition of STAT5 phosphorylation enhanced NPM1 expression. Conversely, NPM1 not only negatively regulated STAT5 phosphorylation but also preserved unphosphorylated STAT5 level. Mechanistically, we show that NPM1 downregulation by P-STAT5 is mediated by impairing the BRCA1-BARD1 ubiquitin ligase, which controls the stability of NPM1. In turn, decreased NPM1 levels led to suppression of p53 expression, resulting in enhanced cell survival. This study reveals a new STAT5 signaling pathway regulating p53 expression via NPM1 and uncovers new therapeutic targets for anticancer treatment in tumors driven by STAT5 signaling.

Signal transducer and activator of transcription 5 (STAT5) is a prominent member of the STAT family, which exists in two highly homologous isoforms, STAT5A and STAT5B. STAT5 phosphorylation at tyrosine 694 (Y694) is essential for cell survival, proliferation, angiogenesis and metastasis in certain cancers of both hematopoietic and non-hematopoietic origin.^1, 2^ STAT5 phosphorylation can be prognostic in patients with breast cancer,^3^ and its overexpression promotes breast cancer formation in mice.^4^ These findings underline the importance of characterizing the downstream targets along the STAT5 signaling pathway and the necessity of identifying regulators of STAT5 phosphorylation. A reciprocally inhibitory relationship has been established between STAT5A and the tyrosine kinase NPM–ALK fusion protein in T-cell lymphoma.^5^ Nucleophosmin (NPM1) is a phosphoprotein involved in many cellular processes, including cell cycle regulation, centrosome duplication and the formation of a complex network with apoptosis-related proteins, such as p53, MDM2 and Arf.^6^ NPM1 can stabilize p53 through direct physical interaction by inhibiting MDM2-mediated p53 ubiquitination.^7, 8^ NPM1 has also been identified as a substrate of BRCA1-BARD1 ubiquitin ligase, which results in its stabilization and localization in the centrosome during cell mitosis to guard against centrosome hyperamplification. ^9^

STAT5 and NPM1 are functionally related as they are both involved in mediating certain biological activities and pathological processes. Both STAT5 and NPM1 are key players in mediating the long-term self-renewal of human stem/progenitor cells.^10, 11^ Moreover, STAT5 and NPM1 abnormalities were separately found in acute myeloid leukemia (AML). Constitutive activation of STAT5 is widely observed in AML, and mutations in NPM1 abrogating its normal function are found in one-third of AML patients.^12, 13^ Furthermore, the oncogenic properties of both STAT5 and NPM1 are tied to their nucleolar localizations. The nucleolar localization of STAT5B is a characteristic feature of the leukemogenic phenotype of chronic myeloid leukemia (CML).^14^ NPM1 mutations in the nucleolar localization signal can cause aberrant accumulation in the cytoplasm and are linked to AML transformation.^13^

Previous studies demonstrated that integration of the Csf2 gene into the genome of transgenic mice carrying the most prevalent phenotype of AML-related NPM1 mutation (NPM^cA/−^) could accelerate the onset of disease.^15^ As Csf2 encodes the cytokine granulocyte macrophage colony-stimulating factor (GM-CSF), a potent activator of STAT5 phosphorylation at Y694,^16^ this finding further links STAT5 activation with NPM1 in tumorigenesis. Moreover, we recently reported that phosphorylated STAT3 physically interacts with NPM1 and transcriptionally enhances NPM1 expression in cancer.^17^ These observations together with the shared functional activities of STAT5 and NPM1 prompted us to investigate the relation between STAT5 and NPM1. Herein we document a reciprocal regulatory relationship and physical interaction between NPM1 and STAT5 and explore their functional significance in regulating p53 expression levels as well as cell survival and apoptotic status. Our results provide novel mechanistic insights into STAT5- and NPM1-mediated activities as well as potential new therapeutic targets.

## Results

### Downregulation of NPM1 is associated with STAT5 phosphorylation at tyrosine 694

**Figure 1:**
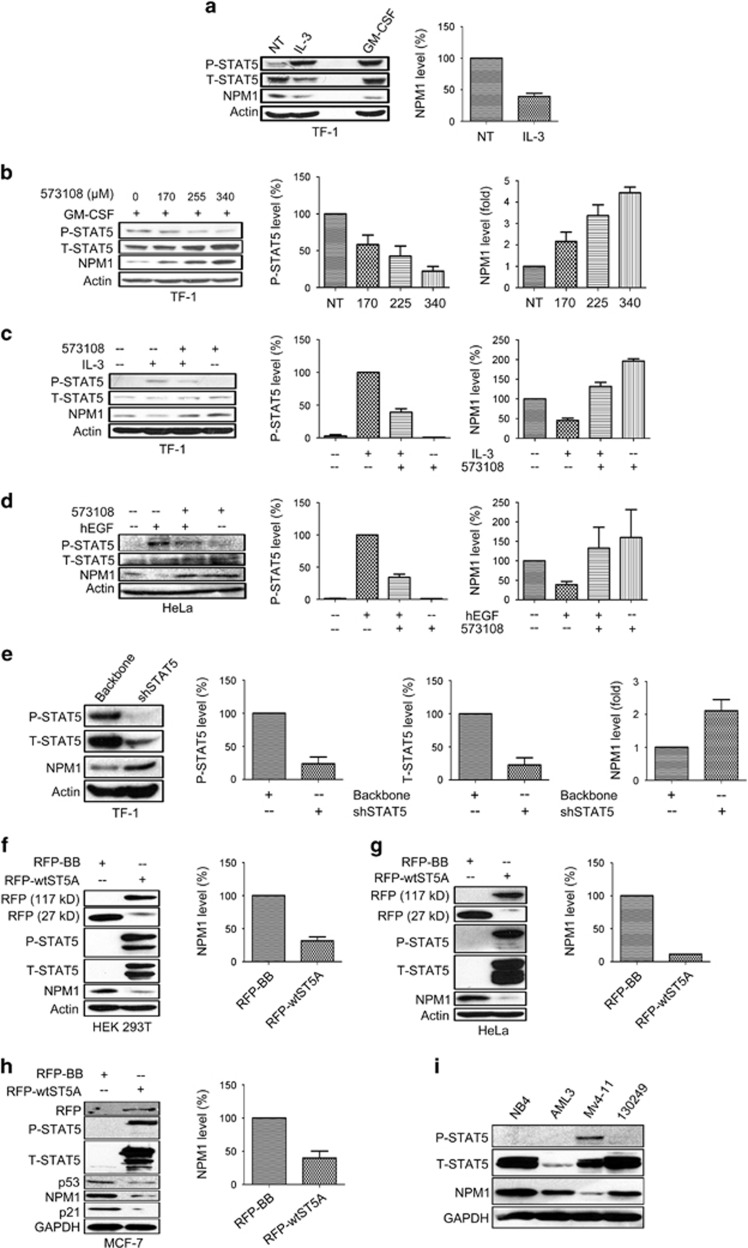
STAT5 phosphorylation at Y694 is associated with decreased NPM1 expression levels. (**a**) 1 × 10^6^ TF-1 cells were deprived of GM-CSF overnight and subsequently stimulated with IL-3 for 3 h at a concentration of 100 ng/ml. Total protein from each cell lysate (50 *μ*g) was loaded in the immunoblotting assay to determine changes in P-STAT5, T-STAT5 and NPM1 expression. TF-1 cells maintained in the presence of GM-CSF (1000 IU/ml) were analyzed as a control. (**b**) 1 × 10^6^ TF-1 cells maintained in GM-CSF supplemented medium were incubated with the inhibitor 573108 at the concentration of 170, 255 and 340 *μ*M, respectively, for 3 h. (**c**) 1 × 10^6^ TF-1 cells deprived of GM-CSF supplement were incubated with the STAT5 inhibitor 573108 at a concentration of 150 *μ*M for 1 h prior to 3 h IL-3 stimulation (100 ng/ml). (**d**) 1 × 10^6^ HeLa cells were stimulated with hEGF (100 ng/ml) for 3 h, preceded or not by a 1 h incubation with the inhibitor 573108 (200 *μ*M). (**e**) TF-1 cells (1 × 10^5^ per condition) maintained in GM-CSF supplemented medium were lentivirally transduced with either shSTAT5 or backbone vector for 72 h using a titer of two TU per cell. (**f** and **g**) HEK 293T, HeLa cells (6 × 10^5^ per well for HEK 293T or 1 × 10^6^ per T-25 culture flask for HeLa cells) were plated on day 0 and transfected with RFP-STAT5A or RFP-backbone vectors on day 1; the transfected cells were harvested and lysed on day 2 for immunoblotting assays on the expression levels of the indicated proteins. (**h**) MCF-7 cells (5 × 10^5^ per well) were transfected with RFP-STAT5A or RFP-backbone vectors for 48 h and harvested and lysed for immunoblot as in the preceding panel. (**i**) A total of 40 *μ*g total protein from each of the four different leukemic cell lysates were loaded on immunoblot assays to measure their STAT5 phosphorylation and NPM1 expression levels. For all experiments shown, densitometry was performed for three independent assays (mean±S.D.) to illustrate the changes of protein expression levels

It has been established that interleukin (IL)-3 can induce STAT5 phosphorylation at Y694 in cell cultures *in vitro*.^1^ Similarly, to maintain persistent STAT5 phosphorylation, GM-CSF is added to the culture medium of human erythroleukemic cell line TF-1.^16^ To investigate whether STAT5 phosphorylation at Y694 regulates NPM1 expression, we induced STAT5 phosphorylation by IL-3 stimulation in TF-1 cells deprived of GM-CSF and observed a significant downregulation of NPM1 coinciding with STAT5 phosphorylation at Y694 (P-STAT5, Figure 1a). This led us to speculate that P-STAT5 negatively regulates NPM1 expression. To test this, we employed three different STAT5 inhibitors, namely, 573108, AC-3-19 and AC-4-130, to specifically inhibit STAT5 phosphorylation at Y694 in TF-1 cells.^18, 19^ The inhibitor treated cells displayed significantly increased NPM1 levels, which was paralleled by decreased levels of P-STAT5 (Figure 1b and Supplementary Figure S1). Moreover, the IL-3-induced NPM1 decrease could be reversed by these inhibitors (Figure 1c and Supplementary Figure S1). These data suggest that P-STAT5 is a negative regulator of NPM1 expression.

As another model, we stimulated HeLa cells with human epidermal growth factor (hEGF), which also induces STAT5 phosphorylation at Y694,^20^ and observed a significant decrease of NPM1 in these cells (Supplementary Figure S2). Moreover, the treatment of hEGF in combination with inhibitor 573108 recovered NPM1 level from the hEGF-induced decrease (Figure 1d).

To rule out potential off-target effect for the inhibitors, we transduced TF-1 cells with a lentiviral vector carrying a STAT5 targeting shRNA (shSTAT5), and the decline in STAT5 phosphorylation was again associated with an NPM1 upregulation (Figure 1e), thus providing direct evidence for the STAT5-mediated NPM1 downregulation. Conversely, we also ectopically expressed an RFP-tagged wild-type STAT5A (RFP-wtSTAT5A) vector in HEK 293T (Supplementary Figure S3), HeLa and MCF-7 cells, none of which display detectable STAT5 phosphorylation at Y694 in immunoblotting assays. In all three cell types, the RFP-wtSTAT5A-expressing cells exhibited significantly downregulated NPM1 levels in comparison with those transfected with backbone vector (Figures 1f–h). Furthermore, HEK 293T and MCF-7 cells transfected with STAT5AY694F mutant vector (RFP-mutSTAT5A) did not show any change in NPM1 expression level (Figures 5c, d and f), highlighting the crucial role of Y694 phosphorylation in downregulating NPM1 expression.

We also investigated the correlation between STAT5 phosphorylation and NPM1 expression level in four different types of AML cells, including the primary cells referred to as ‘130249'. Compared with the other three lines, the Mv4-11 cells exhibiting an evident mutant FLT3-induced STAT5 activation^21^ also displayed significantly lower levels of NPM1 expression (Figure 1i).

Taken together, these data established that STAT5 phosphorylation at Y694 can downregulate NPM1 expression.

### NPM1 has opposite effects on phosphorylated and unphosphorylated STAT5 levels

**Figure 2:**
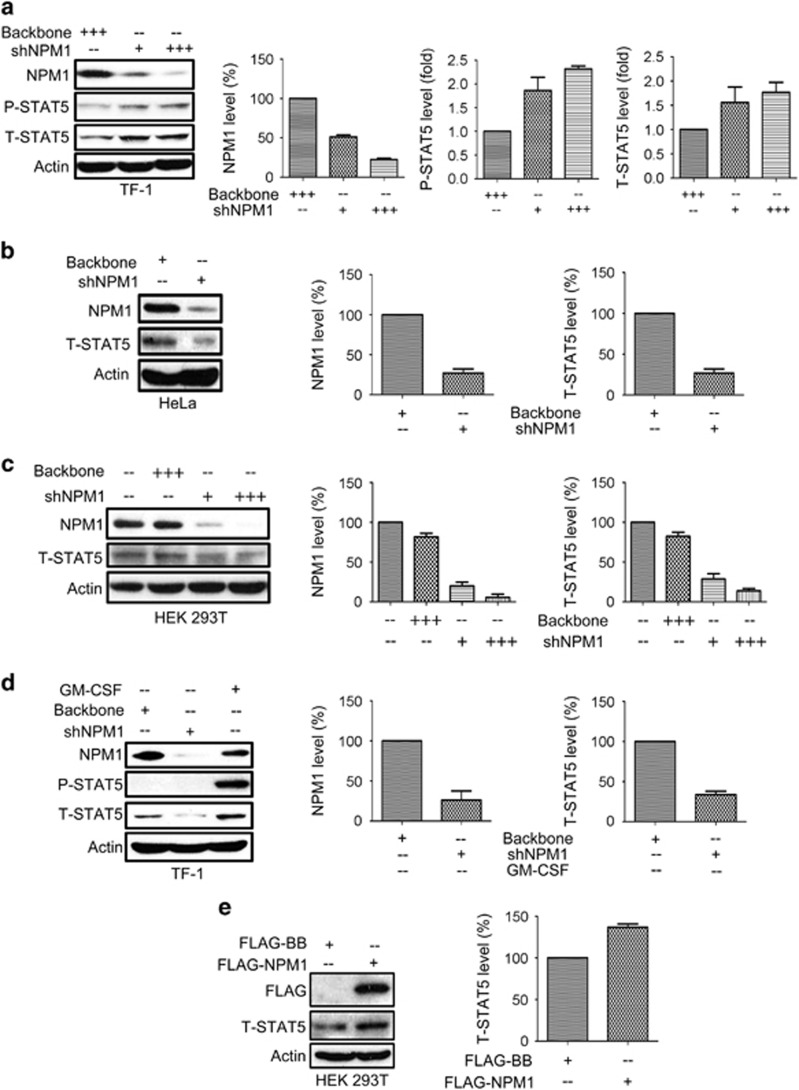
Knockdown of NPM1 enhances P-STAT5 expression but compromises T-STAT5 expression in the circumstance where P-STAT5 is absent. (**a**) 1.5 × 10^5^ TF-1 cells were lentivirally transduced with shNPM1 using a titer of 1–3 TU per cell for 72 h. (**b**) HeLa cells (1.5 × 10^5^ per condition) were transduced with either backbone or shNPM1 at a titer of two TU per cell for 72 h. (**c**) HEK 293T cells (1.5 × 10^5^ per condition) were lentivirally transduced with shNPM1 using viral titers of 1–3 TU per cell for 72 h. NPM1 and T-STAT5 expression were quantified by performing densitometry. (**d**) TF-1 cells (1.5 × 10^5^ per condition) were deprived of GM-CSF and transduced with shNPM1 at viral titer of three TU per cell for 72 h. (**e**) HEK 293T cells (6 × 10^5^ per well) were plated on six-well plate on day 1 and transfected with either FLAG-NPM1 or FLAG-backbone vectors on day1 and then the transfected cells were collected and lysed on day 2 for immunoblotting assays on the protein expression of T-STAT5. For all assays, statistical analysis was performed based on three independent assays and bar graphs represent mean±S.D.

We then asked whether NPM1 can conversely influence STAT5 expression and therefore transduced TF-1 cells with a lentiviral vector carrying shRNA targeting NPM1 (shNPM1). The transduced cells displayed a significant upregulation of P-STAT5 (Figure 2a), indicating a negative influence of NPM1 on STAT5 phosphorylation. Subsequently, we also transduced HeLa and HEK 293T cells that show no detectable P-STAT5 with shNPM1 and observed a significant total STAT5 (T-STAT5) decrease in both lines (Figures 2b and c). These data suggest that NPM1 has distinct effects on P-STAT5 and unphosphorylated STAT5 (U-STAT5). We also transduced the GM-CSF-deprived TF-1 cells with shNPM1 and observed a significant decrease in T-STAT5 in the P-STAT5-negative cells (Figure 2d), confirming that NPM1 maintains the U-STAT5 level. To conversely verify the impact of NPM1 on U-STAT5, we also expressed FLAG-NPM1 vector in HEK 293T cells, and the exogenous NPM1 expression led to an increase in T-STAT5 level (Figure 2e). This validates the role of NPM1 in maintaining U-STAT5. So far, we provided evidence establishing the mutual regulatory relationship between STAT5 and NPM1.

### STAT5 physically interacts with NPM1

**Figure 3:**
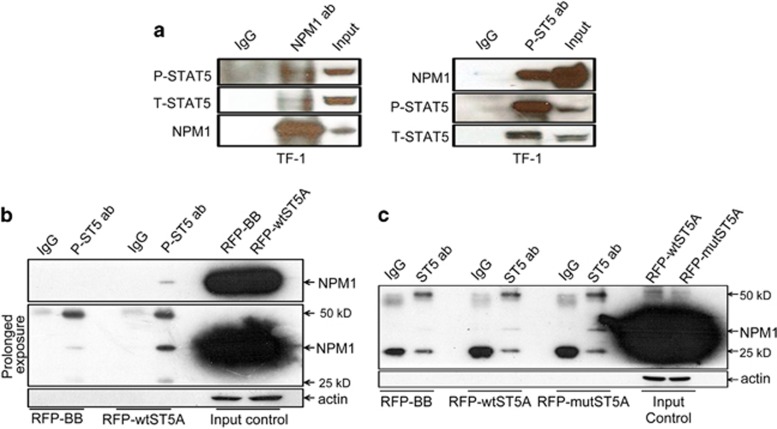
NPM1 physically associates with STAT5. (**a**) Lysates from TF-1 cells (4 × 10^6^ cells per condition) maintained in GM-CSF were immunoprecipitated with monoclonal anti-NPM1 antibody (1 *μ*g per condition) and monoclonal anti-phospho STAT5 antibody (1:200) and probed with the indicated antibodies. (**b**) HEK 293T cells were plated in T-75 culture flasks on day 0 and transfected with either RFP-STAT5A or RFP-backbone vectors at the confluence of 80–90% on day 1, and the transfected cells were harvested and lysed on day 2. Lysate containing 2000 *μ*g total protein was precipitated with 5 *μ*l mouse monoclonal anti-phospho-STAT5 antibody (1:100) or 20 *μ*l mouse IgG sepharose beads, mouse monoclonal anti-NPM1 antibody was used for detection. (**c**) The transfection of RFP-STAT5AY694F mutant vector followed the same procedure as the preceding panel. The lysate carrying 2000 *μ*g total protein was precipitated with rabbit monoclonal anti-STAT5 antibody (1:50) or rabbit IgG XP Isotype control (1:50), mouse monoclonal anti-NPM1 antibody was used for detection

We recently discovered the physical engagement between STAT3 and NPM1 in cancer cells.^17^ Moreover, it was documented that STAT5 physically associates with centrosomal P4.1-associated protein^22^ and that NPM1 regulates centrosome duplication.^23^ These findings prompted us to ask whether a physical interaction exists between STAT5 and NPM1. To address this, we first determined the subcellular localizations of both proteins performing confocal microscopy on TF-1 cells. Although there is a clear overlap between the STAT5 and NPM1 signals in the nuclear region, this in itself is insufficient to argue for their physical interaction (Supplementary Figure S4). We therefore performed immunoprecipitation assays on TF-1 cells. As shown in Figure 3a, STAT5 can be precipitated with anti-NPM1 antibody, and conversely, NPM1 can also be precipitated with anti-P-STAT5 antibody, suggesting a direct interaction between STAT5 and NPM1. Moreover, when the lysate of the HEK 293T cells exogenously expressing RFP-wtSTAT5A vector were precipitated with anti-P-STAT5 antibody, NPM1 was readily detected (Figure 3b). Finally, we precipitated the lysate of HEK 293T cells transfected with RFP-STAT5AY694F mutant vector using anti-T-STAT5 antibody. Again, a clear NPM1 signal was detected, indicating that STAT5 phosphorylation at Y694 is not essential for the physical interaction between STAT5 and NPM1 (Figure 3c). Taken together, we demonstrate that STAT5 and NPM1 can form a physical complex.

### Decreased NPM1 levels upon STAT5 activation are associated with increased protein degradation

**Figure 4:**
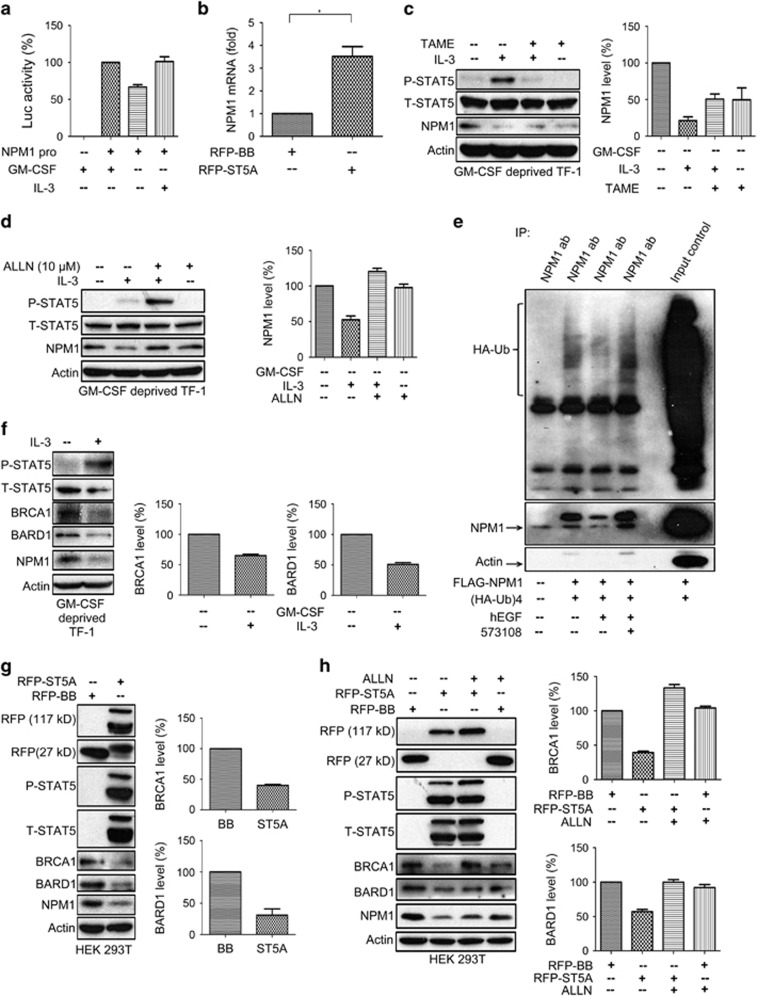
STAT5 phosphorylation downregulates NPM1 by impairing the BRCA1-BARD1 ubiquitin ligase. (**a**) 2.5 × 10^5^ TF-1 cells maintained in GM-CSF supplemented medium were transiently transfected with pGL3-luc-NPM1 promoter vector on day 1 using Lipofectamine 2000 and were deprived of GM-CSF on day 2 for 16 h. The GM-CSF-deprived TF-1 cells were treated with or without IL-3 (100 ng/ml) for 2 h on day 3. Luciferase assays were performed for each experimental condition. (**b**) Quantitative reverse transcriptase-PCR were performed to analyze the relative NPM1 mRNA levels in HEK 293T cells transfected with either RFP-STAT5A vector or backbone control for 48 h. (**c**) 1 × 10^6^ TF-1cells were deprived of GM-CSF and simultaneously treated with TAME hydrochloride (5 mM) for 16 h, followed by 3 h of IL-3 treatment (100 ng/ml). (**d**) 1 × 10^6^ TF-1 cells deprived of GM-CSF overnight were treated with 10 *μ*M ALLN for 1 h prior to IL-3 treatment (100 ng/ml) for 3 h. (**e**) HeLa cells were plated on T-75 culture flasks on day 0 and co-transfected with pcDNA3-FLAG-NPM1 and pcMV-(HA-ubiquitin)_4_ vectors on day 1. The transfected cells were treated with either hEGF (100 ng/ml) alone for 2 h or inhibitor 573108 (225 *μ*M) for 1 h prior to a 3 h hEGF treatment. Cells receiving different treatments were lysed and immunoprecipitated with mouse monoclonal anti-NPM1 antibody (5 *μ*g per condition) and probed with anti-HA antibody to determine the ubiquitin conjugated to NPM1 in each condition. (**f**) 1 × 10^6^ TF-1 cells deprived of GM-CSF overnight were stimulated with IL-3 at the concentration of 100 ng/ml for 3 h. The cell lysate were subjected to immunoblotting assays to determine the expression levels of BRCA1, BARD1 and NPM1. (**g**) HEK 293T cells (6 × 10^5^ per well) were plated in a six-well plate on day 0 and transfected with either RFP-STAT5A or RFP-backbone vectors on day 1. The transfected cells were harvested and lysed for immunoblotting assays on the expression levels of BRCA1, BARD1 and NPM1. (**h**) HEK 293T cells (6 × 10^5^ per well) were plated in a six-well plate on day 0 and transfected with either RFP-STAT5A or RFP-backbone vectors for 6 h, followed by a 1.5 h incubation with ALLN at a concentration of 10 *μ*M. The cells were harvested and lysed on day 2 for immunoblotting assays on the changes in expression level of BRCA1 and BARD1. For all experiments, statistical analysis was based on three independent assays (mean±S.D.). For immunoblotting, one out of the three representative experiments was shown each time

We next investigated the potential mechanism by which P-STAT5 modulates NPM1 expression. We first explored the impact of P-STAT5 on transcriptional activities of NPM1 gene by transfecting cells with the vector containing a NPM1 promoter driving luciferase reporter (pGL3-Luc-NPM1). Compromising STAT5 phosphorylation by either depriving TF-1 cells of GM-CSF or treating HeLa cells with inhibitor 573108 resulted in decreased luciferase activities, whereas stimulation with IL-3 in TF-1 cells or hEGF could significantly upregulate the luciferase activities (Figure 4a and Supplementary Figure S5). To characterize the transcriptional regulation of the NPM1 gene by STAT5 phosphorylation, we performed q-PCR assays on the HEK 293T cells transfected with RFP-STAT5A vector. We observed that the mRNA level of the NPM1 gene in the STAT5A-expressing cells was 3–4-fold higher than that of those transfected with backbone control (Figure 4b). These data indicate that cytokine/growth factor-induced STAT5 phosphorylation at Y694 leads to transcriptional enhancement of NPM1 expression, which appears to be at odds with the finding of P-STAT5-mediated downregulation of NPM1 expression.

Gupta *et al.*^24^ demonstrated that STAT5 phosphorylation downregulates LEF1 expression by enhancing its ubiquitination and degradation. We therefore speculated that P-STAT5 might also accelerate ubiquitination-mediated degradation of NPM1 protein to offset the cytokine/factor-induced NPM1 transcriptional enhancement. To test this hypothesis, we separately exposed GM-CSF-deprived and IL-3-treated TF-1 cells to two protease inhibitors, namely, TAME hydrochloride and the Calpain inhibitor I *N*-acetyl-L-leucyl-L-leucyl-L-norleucinal (ALLN).^25, 26^ As shown in Figures 4c and d, the IL-3-induced NPM1 decrease can be reversed by both inhibitors, indicating that STAT5 phosphorylation downregulates NPM1 expression by inducing proteasome-mediated protein degradation.

We further explored the ubiquitination status of NPM1 under various degrees of STAT5 phosphorylation. For this purpose, we co-transfected HeLa cells with FLAG-NPM1 and HA-tagged ubiquitin (HA-ub)_4_ vectors, treated them with hEGF alone or hEGF in conjunction with the P-STAT5 inhibitor 573108 and then performed *in vitro* ubiquitination assays. The ubiquitin level in hEGF-treated cells was substantially lower than that in those receiving no hEGF treatment. Moreover, the hEGF-induced decrease of ubiquitin could be reversed by the 573108 treatment (Figure 4e). However, it should also be noted that the amount of precipitated NPM1 in each condition also changed along with its corresponding ubiquitin level. Therefore, it is difficult to establish a causal relationship between the change in NPM1-conjugated ubiquitin and the variation in the precipitated NPM1 levels.

To address this ambiguity, we investigated the potential changes in BRCA1 and BARD1 expression upon STAT5 phosphorylation, as NPM1 protein is stabilized by the BRCA1-BARD1 RING heterodimer as their target for ubiquitination.^9^ We first determined the BRCA1 and BARD1 expression level in TF-1 cells treated with IL-3 and found that the IL-3 treatment caused a significant decrease in both BRCA1 and BARD1 expression (Figure 4f). We also assessed their expression levels in the HEK 293T cells transfected with RFP-wtSTAT5A vector and again observed a significant decrease of BRCA1 and BARD1 expressions in these cells (Figure 4g). Furthermore, we treated HEK 293T cells expressing RFP-wtSTAT5A vector with the Calpain inhibitor ALLN and found that NPM1 level was significantly restored along with that of BRCA1 and BARD1 (Figure 4h), establishing that ubiquitination-related proteolysis has a vital role in regulating NPM1 expression. Taken together, our data indicate that STAT5 phosphorylation at Y694 destabilizes NPM1 by impairing BRCA1-BARD1 E3 ubiquitin ligase.

### Regulation of NPM1 expression by STAT5 affects p53 expression

We next wondered about the functional significance of the P-STAT5–NPM1 signaling pathway. NPM1 has an important role in maintaining p53 stability^27^ and has an inhibitory effect on the expression of MDM2 (Supplementary Figure S6),^28^ a major negative regulator of p53. Moreover, STAT5 phosphorylation was inversely related to p53 expression under oncogenic circumstances.^29, 30^ It is therefore conceivable that STAT5 phosphorylation at Y694 suppresses p53 expression by downregulating the NPM1 expression level.

**Figure 5:**
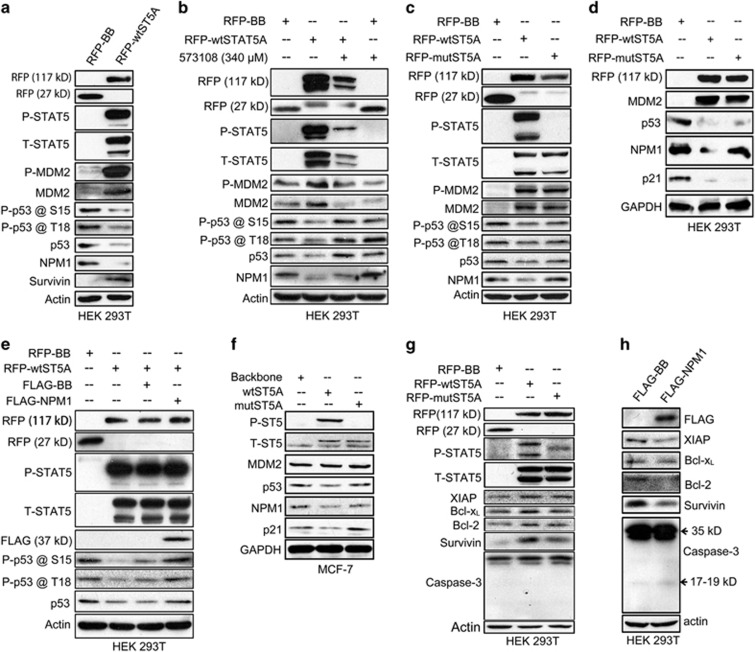
P-STAT5 regulates p53/MDM2 functions and cell survival through NPM1 protein. (**a**) HEK 293T cells (6 × 10^5^ per well) transfected with either RFP-STAT5A or RFP-backbone vectors were harvested at 24 h after transfection, and the cell lysates were subjected to immunoblotting assays for detection of expression levels of p53 and its phosphorylation levels at serine 15 and threonine 18, as well as the expression levels of MDM2 and its phosphorylation level at serine 166. (**b**) Six hours after transfection with either RFP-STAT5A or RFP-backbone vectors, HEK 293T cells (6 × 10^5^ per well) were incubated overnight with inhibitor 573108 at a concentration of 340 *μ*M and then harvested and lysed for immunoblotting assays to examine the expression levels of p53 and MDM2 as well as their relevant phosphorylation levels. (**c** and **d**) HEK 293T cells (6 × 10^5^ per well) were transfected with RFP-STAT5AY694F mutant vector for 24 h, harvested and lysed for immunoblotting assays on the expression levels of MDM2, p53 and p21. (**e**) HEK 293T cells (6 × 10^5^ per well) were co-transfected with RFP-wtSTAT5A and FLAG-NPM1 vectors for 24 h, harvested and lysed for immunoblotting assays on the p53 expression levels. (**f**) MCF-7 cells (2 × 10^6^ per well) were plated on 10 cm plate on day 0 and then lentivirally transduced with pLVX-IRES-mCherry-wtSTAT5 or pLVX-IRES-mCherry-STAT5Y694F on day 1. The cells were harvested 72 h after the transduction and lysed for immunoblotting assays. (**g**) HEK 293T cells (6 × 10^5^ per well) were transfected with either RFP-STAT5A or RFP-backbone vectors for 24 h, collected and lysed for immunoblotting assays on the expression levels of XIAP, Bcl-x_L_, Bcl-2, survivin and caspase-3. (**h**) HEK 293T cells (6 × 10^5^ per well) transfected with FLAG-NPM1 or FLAG-backbone were subjected to immunoblotting assays on the expression levels of the same proteins indicated in the preceding panel. Immunoblotting data in each panel are representative of at least three independent assays

To test this hypothesis,we first measured the p53 expression level in HEK 293T cells transfected with the RFP-wtSTAT5A vector and found a significant decrease in total p53 expression level along with compromised phosphorylation levels at both serine 15 (Supplementary Figure S15) and threonine (Supplementary Table S18) sites (Figure 5a and Supplementary Figures S7A−C). Intriguingly, exogenous expression of STAT5A also led to a significant upregulation in both total MDM2 (T-MDM2) and serine 166 phosphorylated MDM2 (P-MDM2) (Figure 5a and Supplementary Figures S7D and E). To explore the role of STAT5 phosphorylation in regulating p53 expression, we administered the inhibitor 573108 to the HEK 293T cells expressing the RFP-wtSTAT5A vector and observed that inhibition of STAT5 phosphorylation at Y694 reversed p53 downregulation and MDM2 enhancement (Figure 5b and Supplementary Figure S8). We also transfected HEK 293T cells with RFP-STAT5AY694F mutant vector and found that the introduction of Y694F mutation could only mildly downregulate p53 expression levels, although it elicited an evident increase in MDM2 expression levels (Figure 5c and Supplementary Figure S9). These data suggest a vital role for NPM1 in mediating the STAT5-induced decrease in p53 expression. We also verified these findings using another p53 antibody (Santa Cruz Biotechnology). As shown in Figure 5d, transfection with the wild-type STAT5A vector abrogated both NPM1 and p53 expression levels in HEK 293T cells. Although it had little effect on the NPM1 expression, overexpression of the STAT5A Y694F mutant vector significantly reduced p53 expression levels. Moreover, considering that p53 regulates p21 expression and stability, and that p21 acts as a downstream effector of p53 by inducing cell cycle arrest and cellular senescence,^31, 32, 33^ we also measured p21 levels in the transfected cells and observed a concomitant decrease in p21 expression, along with the reduced p53 levels in the HEK 293T cells expressing wild-type STAT5A (Figure 5d). We then co-expressed RFP-wtSTAT5A and FLAG-NPM1 vectors in HEK 293T cells and observed that overexpression of NPM1 could rescue p53 expression from the RFP-wtSTAT5A-mediated decrease (Figure 5e and Supplementary Figure S10), further validating the bridging role of NPM1 between STAT5 phosphorylation and suppressed p53 expression levels.

As the SV40 large T antigen carried by HEK 293T cells may impair p53 function, we decided to expand our findings in MCF-7 cells, which carry a wild-type p53. Even though no change in MDM2 was elicited, STAT5 phosphorylation-induced NPM1 downregulation resulted in a significant decrease in both p53 and p21 expression levels (Figures 1h and 5f). These results even better demonstrate the suppressive effect of STAT5–NPM1 axis upon p53–p21 signaling.

Based on these findings, we speculated that STAT5 signaling might impact on cell survival. To test this, we measured a range of widely recognized pro-survival and apoptotic markers in HEK 293T cells transfected with STAT5 vectors. Cells expressing either wtSTAT5A or the STAT5AY694F mutant displayed elevated expression levels of pro-survival proteins, including XIAP, Bcl-x_L_, Bcl-2 and survivin, although differences were less pronounced for cells expressing the mutant vector (Figure 5g and Supplementary Figure S11). These data suggest that STAT5 influences the expression of molecules implicated in cell survival. Finally, we also assessed the effect of NPM1 on cell survival and apoptosis by transfecting HEK 293T cells with the FLAG-NPM1 vector. Overexpression of NPM1 not only resulted in a decrease in the expression of pro-survival proteins, such as XIAP, Bcl-x_L_, Bcl-2 and survivin, but also significantly increased caspase-3 cleavage (Figure 5h and Supplementary Figure S12), revealing that NPM1 may possess pro-apoptotic properties.

## Discussion

**Figure 6:**
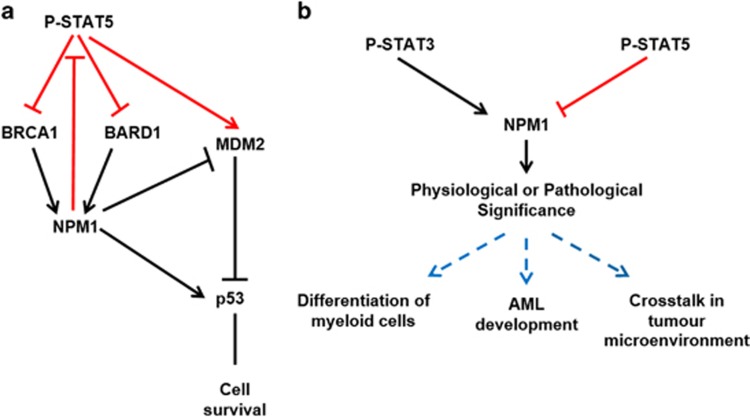
Models for the STAT5 phosphorylation-induced signal transduction through NPM1 protein and the potential physiological and pathological significance of cytokine-induced STAT-NPM1 signal pathways. (**a**) Schematic representation of the mechanism as well as functional relevance of the signalings activated by STAT5 phosphorylation. The signal pathways identified in the present study are indicated in orange, whereas the previously known pathways in black. (**b**) Schematic representation of our proposed STAT–NPM1 axis (STAT5–NPM1 signaling in orange, STAT3-NPM1 signaling in black) and its potential functions in biological activities and tumorigenesis (in blue)

In the present study, we document a mutually regulatory relationship between STAT5 and NPM1. We demonstrate that P-STAT5-mediated downregulation of NPM1 expression is due to impaired ubiquitination by the BRCA1-BARD1 RING complex and that through the decrease of NPM P-STAT5 modulates p53 to execute its pro-survival effects (Figure 6a).

In contrast to our previous finding that STAT3 can transcriptionally enhance NPM1 expression,^17^ STAT5 activation at Y694 resulted in a significant decrease in NPM1 expression. Moreover, unlike the critical role of phosphorylation at Y705 in fostering physical interaction between STAT3 and NPM1, phosphorylation at Y694 is not essential for STAT5 and NPM1 interaction. Opposing effects between STAT3 and STAT5 have been observed on several occasions. For instance, IL-6-induced STAT3 activation can promote T helper 17 (Th17) cell differentiation by positively regulating the transcription factor ROR*γ*t as well as IL-17 expression, whereas IL-2-mediated STAT5 activation disrupts Th17 cell development by constraining IL-17 expression.^34^ Furthermore, constitutive STAT5 activation is dominant over constitutively active STAT3 in certain types of breast cancer cells and antagonizes the positive regulation of STAT3 upon protein BCL6, which is a critical factor in mammary tumorigenesis.^35, 36^

Our study unveils, for the first time, the significant role of NPM1 in mediating the effect of STAT5 on p53. NPM1 is known to have important roles in maintaining p53 stability and regulating its transcriptional activation.^27^ Thus the effect of STAT5 on NPM1 can compromise p53 expression. Moreover, our finding also provides an explanation for the observation that constitutive STAT5 activation coincides with functional loss of p53 in B-cell lymphoma/leukemia.^30^ It was also demonstrated that knockdown of STAT5A in CML hematopoietic progenitors could dramatically increase the p53 expression level,^29^ which is in line with our data. However, it was also noted that constitutively active STAT5 can facilitate cellular senescence in a p53-dependent manner and that the presence of the p53 pathway ensures a robust tumor-suppressing capability to prevent cellular transformation.^37, 38^ Thus the effect of STAT5 on p53 expression is likely context dependent.

The STAT5–NPM1–p53 axis we identified sheds new light on the mechanism underlying the cytokine-mediated rescue of p53-dependent apoptosis. Quelle *et al.*^39^ first observed that IL-3 treatment could rescue *γ*-irradiation-induced cell apoptosis and further unveiled that IL-3-induced JAK signaling has a critical role in suppressing the p53-dependent apoptosis by enhancing Bcl-2 and Bcl-x_L_. The discovery of STAT5–NPM1–p53 axis fills the gap between the activation of JAK signaling and its biological relevance in cell survival, revealing a possible molecular basis for the cell-surviving effect of cytokine-induced JAK signaling. Furthermore, we demonstrated that exogenous expression of STAT5A could upregulate a range of antiapoptotic or cell survival proteins, including XIAP, survivin, Bcl-x_L_ and Bcl-2. These data add to the accumulating evidence that STAT5 regulates the expression of cell survival proteins in cancer cells.^40, 41, 42^

The identification of NPM1 as a downstream player upon GM-CSF/IL-3/hEGF-induced STAT5 signal pathway may deepen the understanding of the role of NPM1 in tumorigenesis. The cytoplasmic-localized NPM1 mutants (NPMc^+^) have a significant role in the development of AML.^13^ Moreover, integration of the GM-CSF encoding Csf2 gene into the genome of NPMc^+^ transgenic mice could accelerate the onset of AML.^15^ In light of the GM-CSF-induced P-STAT5–NPM1 signaling pathway, the increased GM-CSF levels resulting from aberrant Csf2 expression are likely to suppress NPM1 expression level in leukemic cells. Therefore, the synergistic effects of NPMc^+^ expression and Csf2 in causing AML indicate that both aberrant cytoplasmic localization and decreased expression of NPM1 protein are required for the leukemogenesis of AML. Thus NPM1 may be perceived as a cancer suppressor. The putative role of GM-CSF-activated STAT5–NPM1 signaling pathway in AML development should therefore be further investigated.

The GM-CSF/IL-3/hEGF-induced STAT5 signaling pathway and the IL-6/IFN-*α*-induced STAT3 signaling pathway converge at NPM1,^17^ suggesting that NPM1 is likely to be a hub for a complex cytokine–STAT network. For instance, GM-CSF and IL-3 direct the differentiation of common myeloid progenitor into functionally mature myeloid cells.^43, 44, 45^ It would be intriguing to characterize the potential role of GM-CSF/IL-3-induced P-STAT5–NPM1 pathway in governing the development of myelogenic cells (Figure 6b). Moreover, a vast array of pro-inflammatory factors including the cytokines mentioned above can be produced by myeloid-derived stromal cells to conduct the crosstalk between tumor microenvironment and cancer cells. This is well exemplified in IL-6/JAK/STAT3 pathway-mediated cancer inflammation in colorectal cancer: under the transcriptional drive of NF-*κ*B, IL-6 is produced by bone marrow-derived myeloid cells and then activates STAT3 in epithelial cells from which tumor arises.^46^ Furthermore, in the microenvironment of breast cancer, GM-CSF has important roles in both inducing epithelial–mesenchymal transition and contributing to the accumulation of myeloid-derived suppressor cells.^47, 48^ As NPM1 was identified as a downstream effector of both IL-6–STAT3 and GM-CSF–STAT5 signal pathways, the potential role of NPM1 in mediating crosstalk between cancer and its microenvironment warrants further explorations (Figure 6b).

## Materials and Methods

### Cell culture

TF-1 human erythroleukemic cells, HEK 293T, MCF-7 and Mv4-11 cells (biphenotypic B myelomonocytic leukemia) were maintained in the RPMI-1640 medium (Gibco, Paisley, UK) supplemented with 10% fetal bovine serum and penicillin and streptomycin as well as HEPES. However, the TF-1 culture medium was also supplemented with GM-CSF (1000 IU/ml). HeLa cervical cancer cells were maintained in DMEM medium (Gibco) supplemented with 10% fetal bovine serum and penicillin and streptomycin. NB4 and AML3 leukemic cells were maintained in MEM-Alpha medium (Sigma-Aldrich, St. Louis, MO, USA) supplemented with 10% fetal bovine serum and penicillin and streptomycin.

### Antibodies and reagents

For immunoblotting analysis, antibodies against phospho-STAT5 (Y694, 9356) and total STAT5 (9363), p53 (2524), Phospho-p53 (Thr18, 2529), Phospho-p53 (Ser15, 9284), Phospho-MDM2 (Ser166), BRCA1 (9025), Caspase-3 (9662), XIAP (2042), Bcl-2 (4223), Bcl-x_L_ (2762), anti-FLAG (8146) and HA-Tag (2367) were purchased from Cell Signaling (Beverly, MA, USA). Antibodies against RFP (RF5R), BARD1 (PA1-84781) were purchased from ThermoFisher (Rockford, IL, USA), MDM2 antibody (33-7100) was purchased from Invitrogen (Frederick, MD, USA) and NPM1 antibody (ab24412) was purchased from Abcam (Cambridge, UK). Antibodies against GAPDH (FL-335, sc-25778), p21 (C-19, sc-397) and p53 (FL-393, sc-6243) were purchased from Santa Cruz Biotechnology (Dallas, TX, USA). For immunofluorescence analysis, NPM1 antibody (32-5200, Invitrogen, Frederick, MD, USA), Phospho-STAT5 (9351, Cell Signaling) and total STAT5 (9358, Cell Signaling) were applied as primary antibodies. Alexa Fluor 488 anti-mouse IgG (A31572, Invitrogen, Eugene, OR, USA) and Alexa Fluor 555 anti-rabbit IgG (A21202, Invitrogen, Eugene, OR, USA) were used as secondary antibodies in the immunofluorescence assays. Monoclonal antibodies, including NPM1 antibody (32-5200, Invitrogen, Carlsbad, CA, USA), Phospho-STAT5 antibody (9356, Cell Signaling) and STAT5 (9358, Cell Signaling) were applied in the immunoprecipitation assays. STAT5 inhibitor 573108 was purchased from Calbiochem (Darmstadt, Germany). Cytokines IL-3, GM-CSF and proteasome inhibitors *Nα*-*p*-Tosyl-L-arginine methyl ester hydrochloride (TAME hydrochloride), a competitive ubiquitin ligase anaphase-promoting complex/cyclosome (APC/C) inhibitor and Calpain Inhibitor I ALLN, an inhibitor of Calpain I and II, and cathepsin B and L were purchased from Sigma-Aldrich (St. Louis, MO, USA); hEGF was obtained from Cell Signaling.

### Western blotting

Cells were equalized (as indicated in each relevant panel), suspended in ice-cold Nonidet P-40 lysis buffer supplemented with phosphatase and protease inhibitor cocktail tablets (Roche, Mannheim, Germany). The lysate protein concentration was measured using the Bradford assay (Bio-Rad, Hercules, CA, USA). For each lane on the western blotting, an equal amount of total protein was denatured and separated with 10% SDS-PAGE gel, followed by transfer to a PVDF membrane. Both primary and secondary antibodies (GE Healthcare, Little Chalfont, UK) were applied at the dilutions recommended by the manufacturers. The primary antibodies were incubated overnight at 4 °C. The densitometry was performed using the ImageJ software (NIH, Bethesda, MD, USA). Quantifications of at least three independent experiments were shown as histograms using the software GraphPad Prism5 (La Jolla, CA, USA).

### Lentiviral transduction

The shSTAT5 and shNPM1 vectors were kind gifts from Dr. I Dusanter-Fourt of the Institute Cochin and Dr. D Herlyn of the Wistar Instistute, respectively, and have been previously described.^49, 50^ Wild-type and mutant STAT5A were subcloned into pLVX-IRES-mCherry vector using XhoI and XBaI restriction enzyme sites. The production of lentiviral vectors and their subsequent characterization were performed as previously described.^17^ Briefly, for the generation of lentiviral vectors, HEK 293T cells were plated at 15 × 10^6^ cells per 175 cm^2^ and were transfected on the following day with 15, 30 and 45 *μ*g of plasmids encoding the envelope glycoprotein VSV-G, gag/pol and shRNA, respectively, using polyethyleneimine (Polysciences, Eppelheim, Germany). The supernatant containing lentiviral particles were harvested during the following 3 days. To characterize the viral titers, two different methodologies were employed for shSTAT5 and shNPM1 encoding lentiviral vectors. For the GFP encoding shSTAT5 vector, 10^5^ HEK 293T cells were plated in six-well plates and transduced with viral supernatants at twofold serial dilutions ranging from 1/2 to 1/64 in a total volume of 2 ml medium. Three days later, the percentage of GFP^+^ cells was determined by flow cytometry. The following formula was used to calculate the viral titer: Titer=((*F* × Cn)/*V*) × DF (*F*: the frequency of GFP-positive cells determined by flow cytometry; Cn: the total number of target cells infected; *V*: the volume of the inoculum; DF: the viral dilution factor). For the non-GFP-expressing shNPM1 vectors, the colorimetric reverse transcriptase (RT) assay (Roche, Vilvoorde, Germany) was applied. Comparison of the RT content with the titer of VSV-G pseudotyped lentiviral vectors reveals that 1 ng RT correlated with 2.5 × 10^4^ transduction unit (TU).

Target cells were infected by adding the virus-containing supernatant in the presence of protamine sulfate (10 *μ*g/ml, Sigma-Aldrich, Shinagawa, Japan) at the viral titers indicated in each relevant figure. Transduced cells were analyzed for expression 72 h after transduction by performing immunoblotting.

### Immunofluorescence

TF-1 cells were collected on histogrip (Invitrogen, Frederick, MD, USA) coated slides by cytospin (Shandon Cytospin 3, Pittsburgh, PA, USA), were fixed with 4% paraformaldehyde for 15 min at room temperature (RT) and permeabilized in 0.1% Triton X-100 in PBS for 10 min at RT, followed by blocking with 10% donkey serum (Sigma-Aldrich, St. Louis, MO, USA) in PBS for 1 h at RT. Primary antibodies were applied at the manufacturer's recommended concentrations, followed by overnight incubation in a humidified chamber at 4 °C. Fluorophore-conjugated secondary antibodies were applied at the dilution of 1:500 and incubated for 2 h at RT. The cells were mounted with Prolong Gold antifade reagent with DAPI (Invitrogen, Eugene, OR, USA) for nuclear counterstaining. Images were captured at a resolution of 2048 × 2048 using a Carl Zeiss LSM 780 (Carl Zeiss, Jena, Germany) confocal microscope with a × 40 Plan objective lens. The LSM Image Browser version 4.2.0.121 (Carl Zeiss MicroImaging, Jena, Germany) was used to analyze the microscopic slides.

### Cell transfections

The RFP-tagged wild-type STAT5A and STAT5Y694F mutant vectors were kind gifts from Dr. Willlis X Li^51^; the pcDNA3-FLAG-tagged NPM1 and pcMV-(HA-ubiquitin)_4_ vectors were generous gifts from Dr. T Ohta.^9^ HEK 293T or HeLa cells were plated on day 0 and transfected with 2.5 *μ*g vector employing 7.5 *μ*l Lipofectamine 2000 (Life Technology, Carlsbad, CA, USA) on day 1. Cells were harvested and lysed 24 h after transfection on day 2, and immunoblotting assays were performed as described above.

### Immunoprecipitation assays

Cell pellets were lysed in RIPA buffer (150 mM NaCl, 1% Nonidet P-40, 1 mM EDTA, 0.5% sodium Deoxycholate, 0.1% SDS, 20 mM Tris HCl, pH 7.5) supplemented with phosphatase and protease inhibitors (Roche, Mannheim, Germany). Cell lysates were incubated with different precipitating antibodies at the dilutions indicated in the relevant figure legend for 4 h at 4 °C, followed by addition of 100 *μ*l protein G sepharose beads slurry (GE Healthcare, Uppsala, Sweden). The mixture was incubated overnight at 4 °C in constant agitation. The beads were washed with RIPA buffer and then denatured with 2 × loading buffer (4% SDS, 10% 2-mercaptoethanol, 20% glycerol, 0.004% bromophenol blue, 0.125 M Tris-HCl, pH 6.8) in a boiling water bath. For all cell lysates, sepharose beads-conjugated mouse IgG (3420S, Cell Signaling) or Rabbit (DA1E) IgG XP Isotype (3900, Cell Signaling) was incubated as described above and used as a control.

HEK 293T cells grown to 80–90% confluence in T-75 culture flasks were transfected with 10 *μ*g RFP-tagged wtSTAT5A or STAT5AY694F mutant vector using 25 *μ*l Lipofectamine 2000. The transfected cells were harvested and lysed in 500 *μ*l RIPA buffer, and each cell lysate containing 2 mg total protein was precipitated overnight with mouse monoclonal anti-P-STAT5 or rabbit monoclonal anti-T-STAT5 antibody using 100 *μ*l protein G sepharose beads slurry at 4 °C under rotary agitation.

### RT-PCR

RNA was isolated using the EZ-10 DNAaway RNA Minipreps Kit (Markham, ON, Canada) and reverse-transcribed using the SuperScript IV Reverse Transcriptase (Invitrogen, Life Technologies, Carlsbad, CA, USA) according to the manufacturers' instructions. Primers were ordered from Invitrogen Life Technologies (Pleasanton, CA, USA). Quantitative RT-PCR was performed on the LightCycler 480 Instrument II (Roche Diagnostics GmbH, Mannheim, Germany) using triplicate cDNA templates with the PerfeCTa SYBR Green FastMix (Quanta Biosciences, Beverly, MA, USA) according to the manufacturer's instructions. Reaction conditions were as follows: 20 s at 50 °C, 10 min at 95 °C, and 40 cycles of 15 s at 95 °C and 1 min at 60 °C. The RPLPO was used as the housekeeping gene for equalization and determination of the relative mRNA expression, which was determined by the 2^−ΔΔCT^ method. All data were normalized to the backbone controls. Results shown are the means±S.D. of three independent experiments with two technical replicates. Primer sequences used are listed in Supplementary Table S1.

### Ubiquitination assays

In all, 4 × 10^6^ HeLa cells were plated on T-75 culture flasks on day 0. When the cells reached 80–90% confluence on day 1, equal amount of 15 *μ*g FLAG-NPM1 and pcMV-(HA-ubiquitin)_4_ vectors were co-transfected into HeLa cells employing 75 *μ*l Lipofectamine 2000. The transfected cells were treated with hEGF (100 ng/ml) for 2 h in combination with or without a pretreatment of 1 h with inhibitor 573108 (255 *μ*M) 24 h after the transfection. The cells were harvested and lysed in 500 *μ*l RIPA buffer. NPM1 protein was precipitated from lysate containing 1 mg of total protein by overnight incubation with 10 *μ*g mouse monoclonal anti-NPM1 antibody (32-5200, Invitrogen, Frederick, MD, USA) and 100 *μ*l protein G sepharose beads slurry (GE Healthcare, Uppsala, Sweden) at 4 °C under rotary agitation. The beads were rinsed five times with RIPA buffer and boiled in 30 *μ*l loading buffer for 5 min to denature and separate the precipitated protein. The supernatants were loaded and separated on 10% SDS-PAGE gel following the same protocol as described in the western blotting part. Anti-HA antibody was used for the detection of the ubiquitin conjugated to NPM1 protein.

### Luciferase assays

The pGL3-Luc-NPM1 promoter was a generous gift from Dr. Q Pang of Cincinnati Children's Hospital.^52^ TF-1 cells maintained in GM-CSF supplemented RPMI medium were seeded in a six-well plate at 2.5 × 10^5^ cells per well on day 0 and transfected with 1 *μ*g pGL3-Luc-NPM1 promoter vector using 3 *μ*l Lipofectamine 2000 on day 1. The transfected cells were deprived of GM-CSF supplement for 16 h on day 2, then stimulated with IL-3 (100 ng/ml) for 2 h on day 3, harvested and analyzed using the Luciferase Reporter Assay System (Promega, Madison, WI, USA) using SpectraMax M3 Multi-Mode Microplate Reader and its analytical software SoftMax Pro (Molecular Devices, Sunnyvale, CA, USA). HeLa cells were plated in a six-well plate at 3 × 10^5^ cells per well on day 0 and transfected with 2.5 *μ*g pGL3-Luc-NPM1 promoter vectors using 7.5 *μ*l Lipofectamine 2000 on day 1. The cells were treated with hEGF (100 ng/ml) for 2 h in combination with or without 1 h pretreatment with inhibitor 573108 (255 *μ*M) 24 h after transfection on day 2. The cells were then harvested and subjected to the Luciferase Reporter Assay.

### Statistics

Paired Student's *t*-tests (two-tailed, equal variance) and one-way ANOVA were performed to analyze all the densitometry data. One-way ANOVA was performed to analyze the data from luciferase assays. Both types of statistical analyses were performed with the software GraphPad Prism5 (La Jolla, CA, USA). *P*-values of <0.05 were considered significant.
