# Supplementary figures and images for: Phosphorylated STAT5 regulates p53 expression via BRCA1/BARD1-NPM1 and MDM2
Source: Cell Death Dis. 2016 Dec 22;7(12):e2560–. doi: 10.1038/cddis.2016.430 (PMC5260985; doi:10.1038/cddis.2016.430)

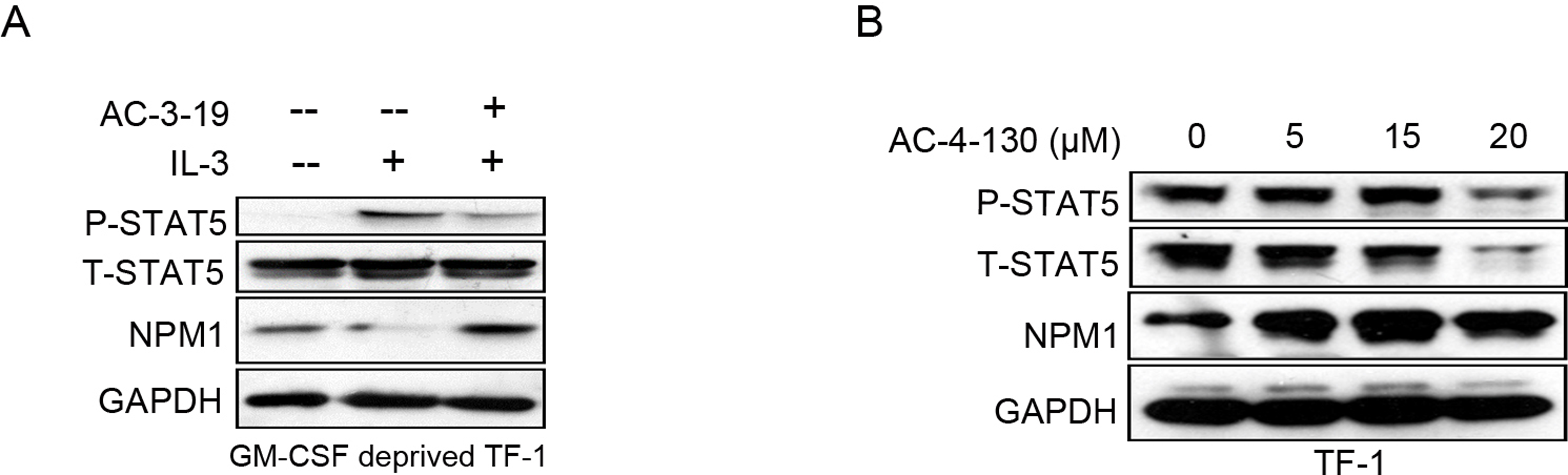

Supplement: Supplementary Figure 1 [file cddis2016430x1.tif]

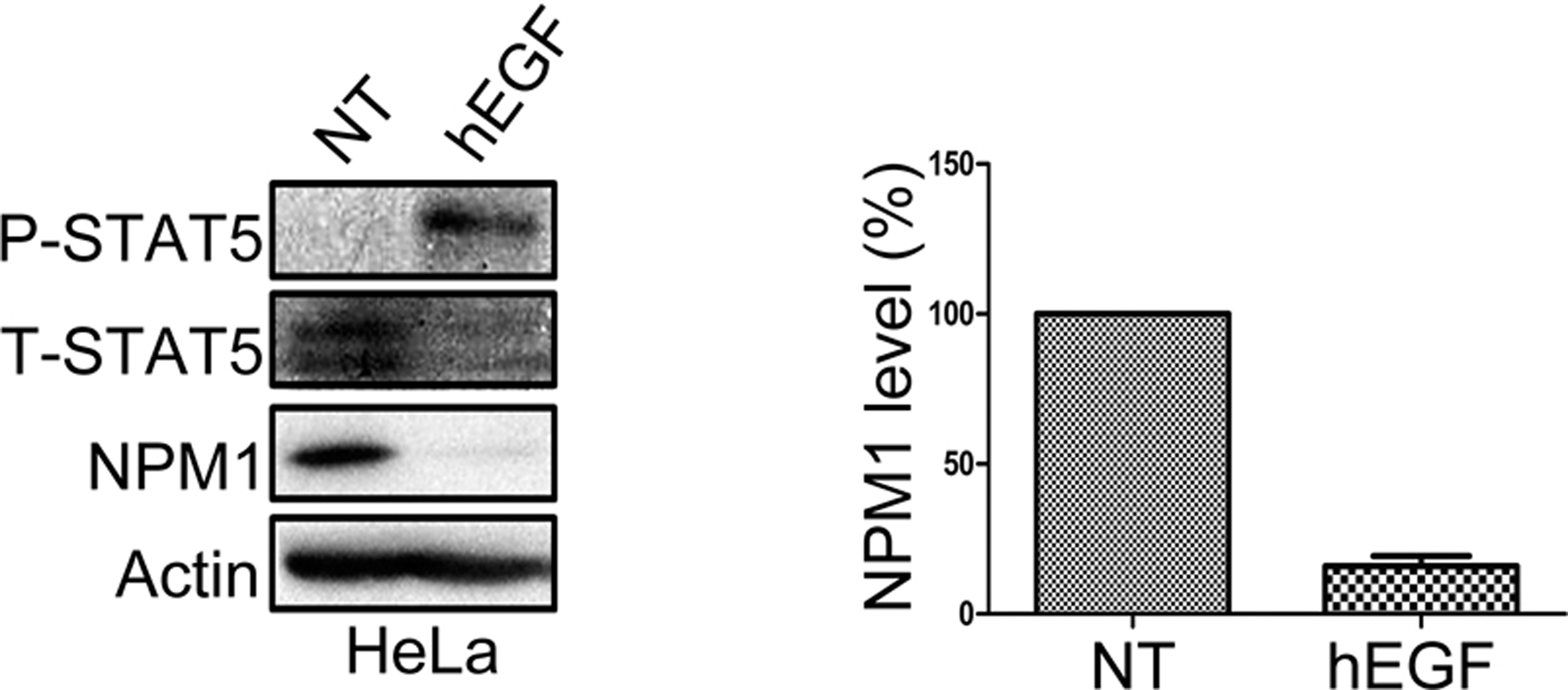

Supplement: Supplementary Figure 2 [file cddis2016430x2.tif]

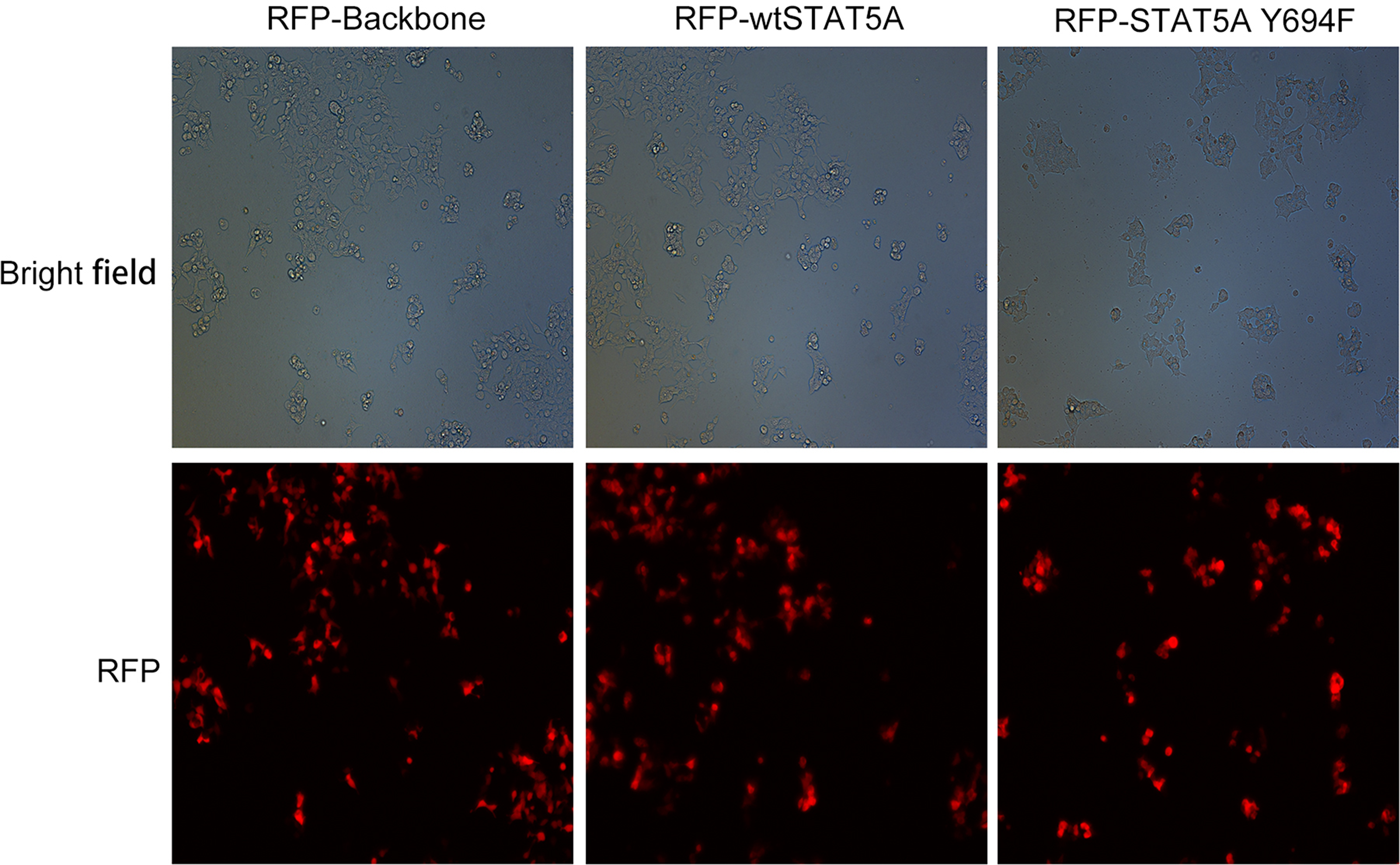

Supplement: Supplementary Figure 3 [file cddis2016430x3.tif]

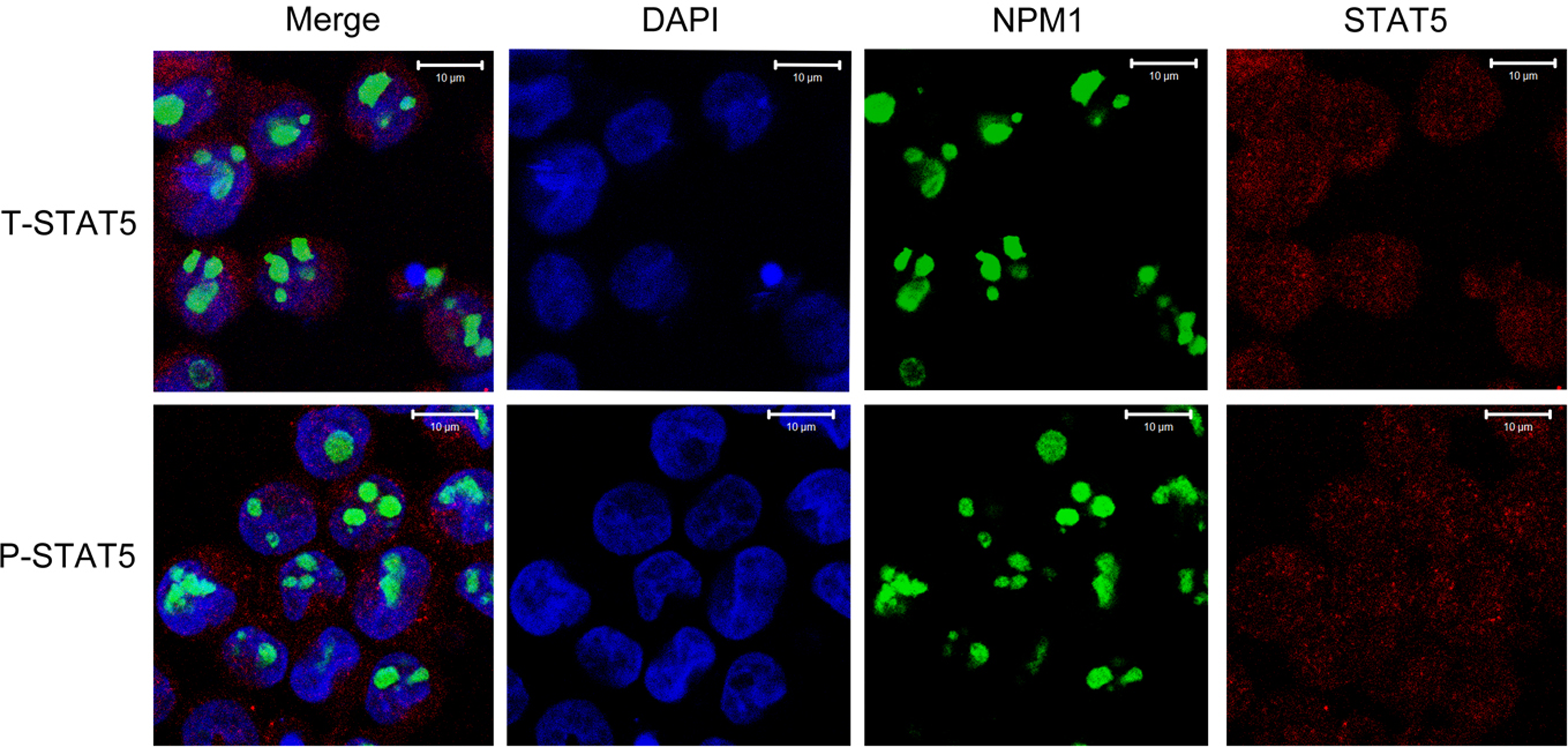

Supplement: Supplementary Figure 4 [file cddis2016430x4.tif]

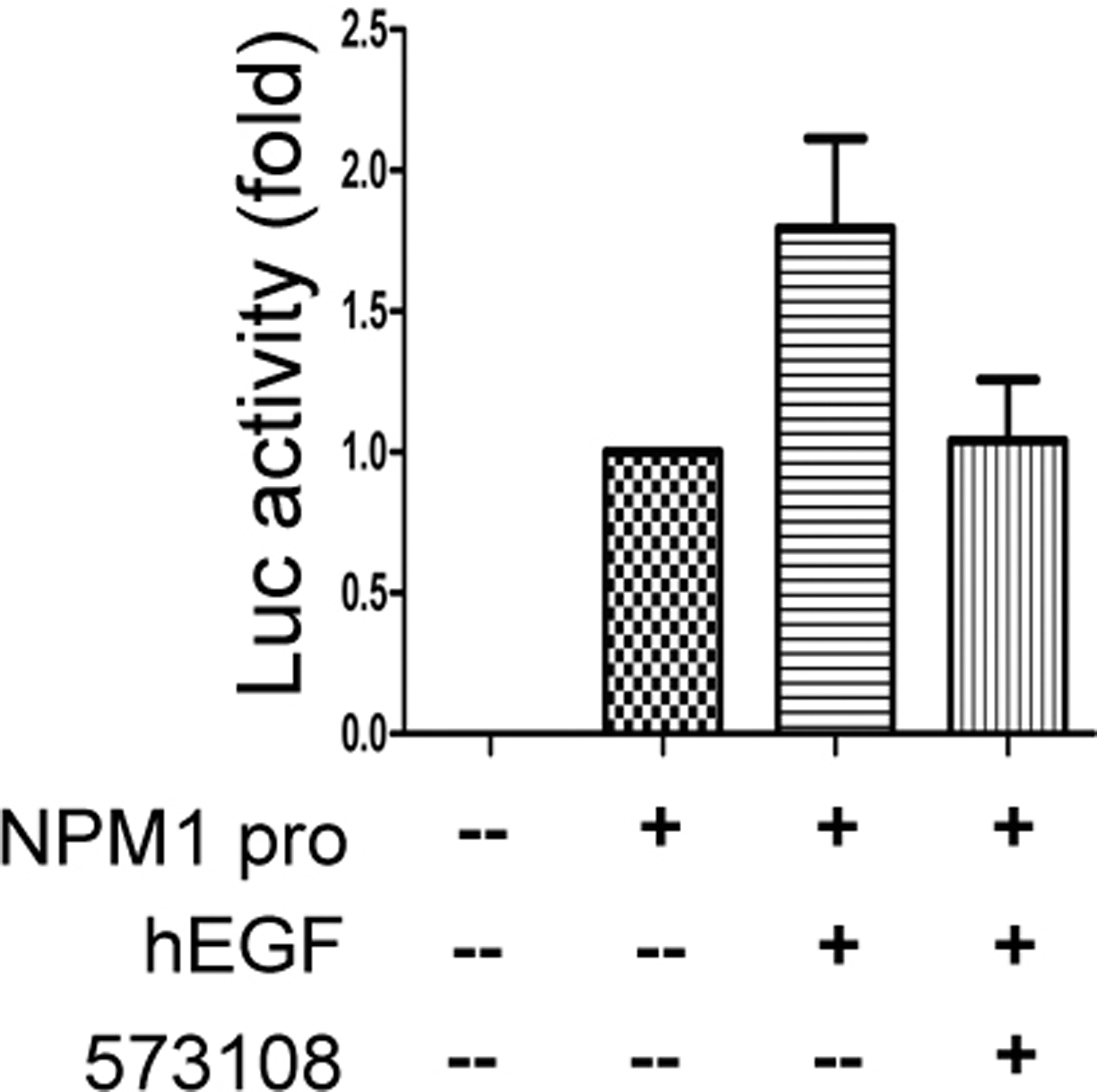

Supplement: Supplementary Figure 5 [file cddis2016430x5.tif]

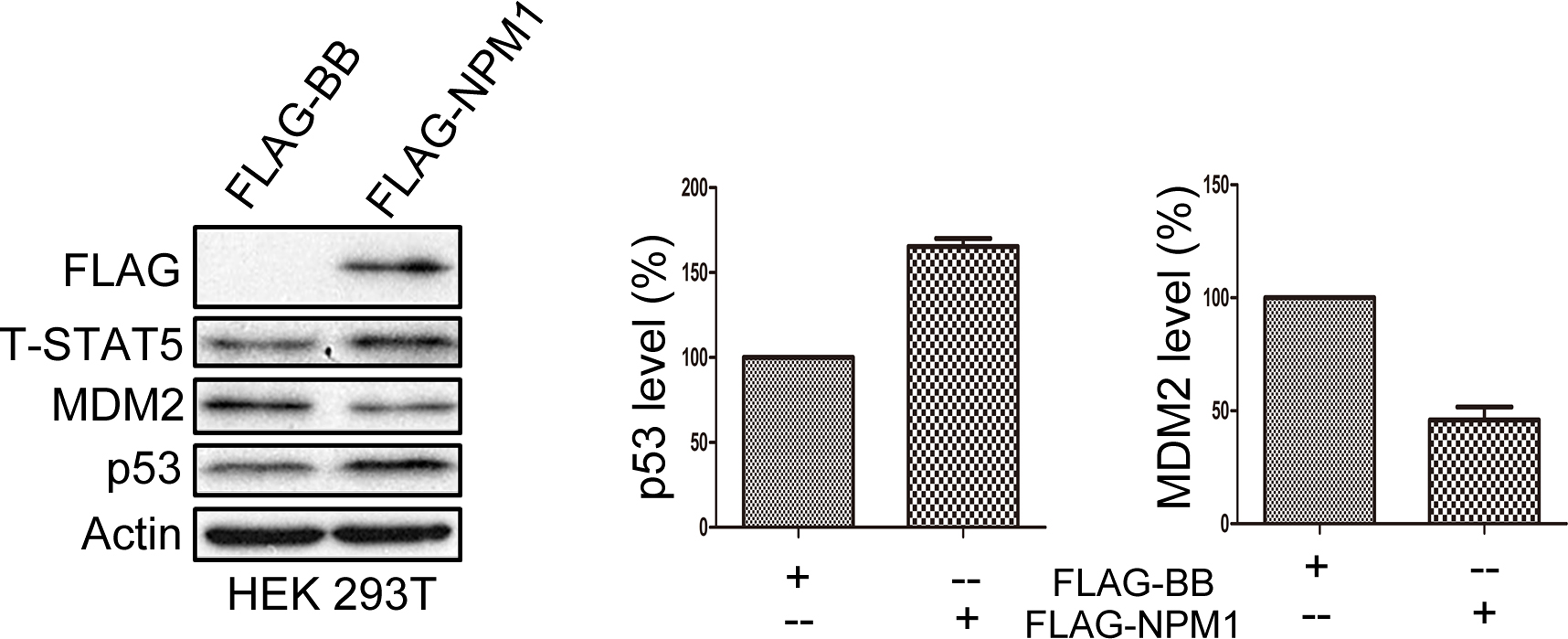

Supplement: Supplementary Figure 6 [file cddis2016430x6.tif]

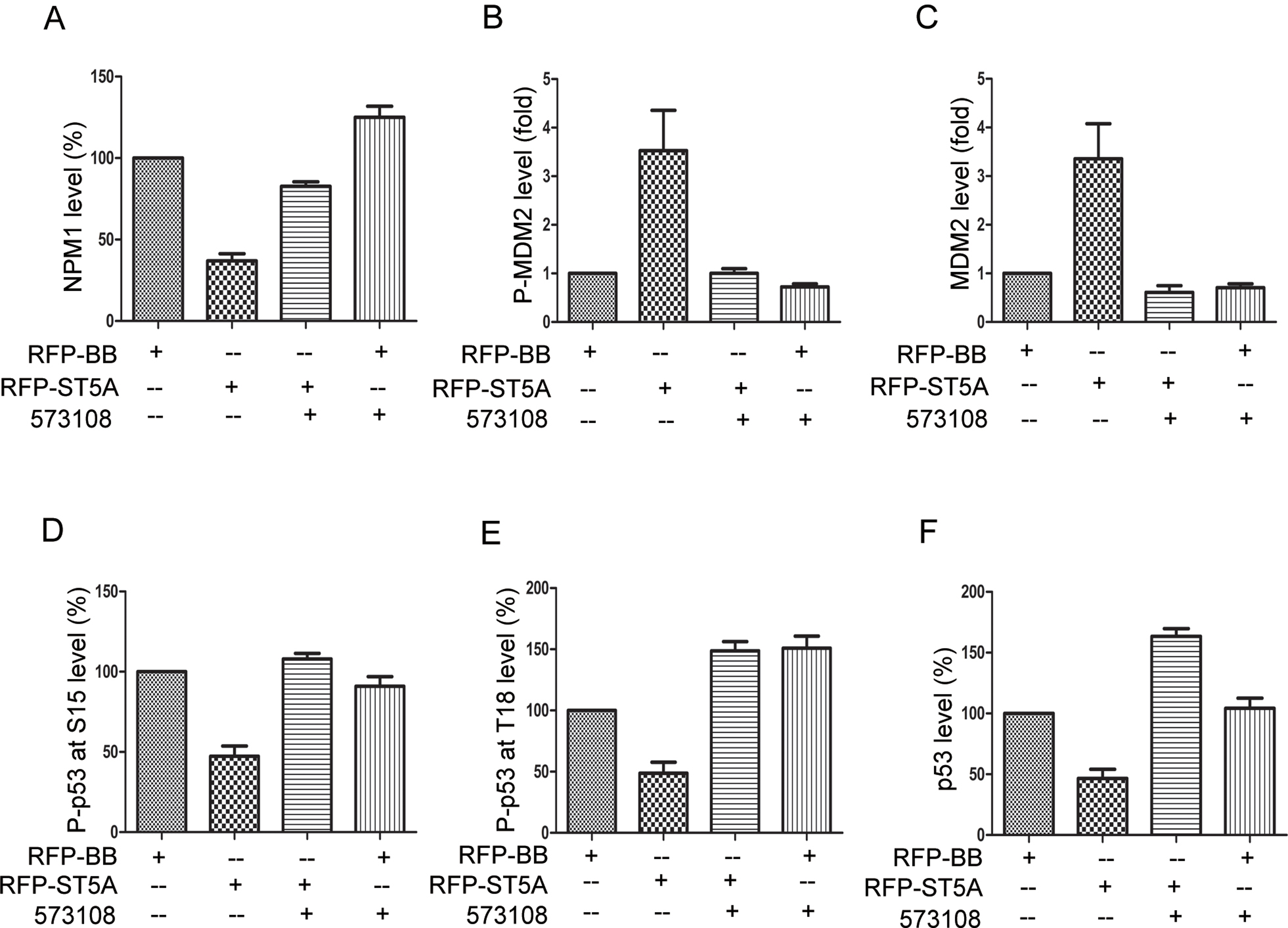

Supplement: Supplementary Figure 8 [file cddis2016430x8.tif]

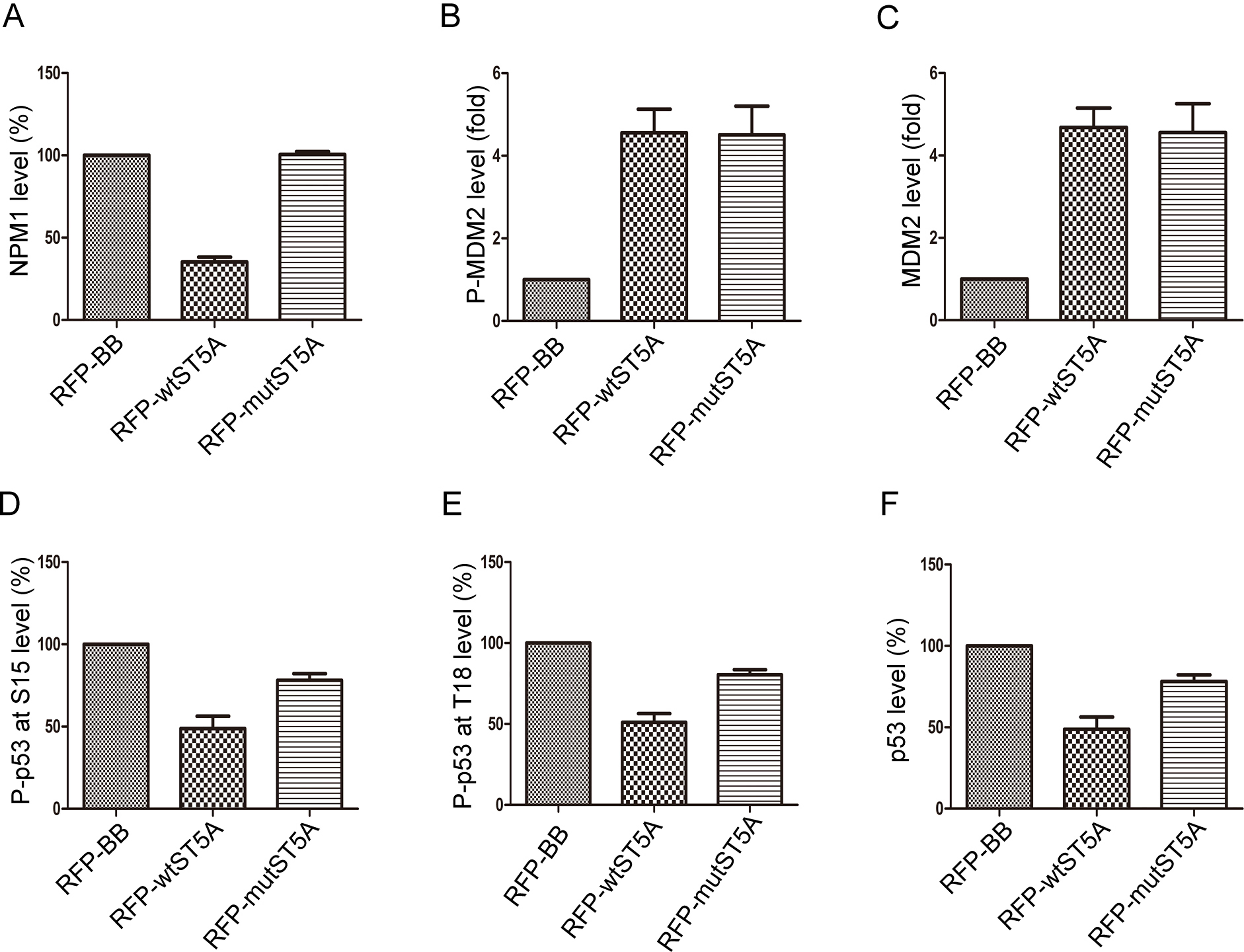

Supplement: Supplementary Figure 9 [file cddis2016430x9.tif]

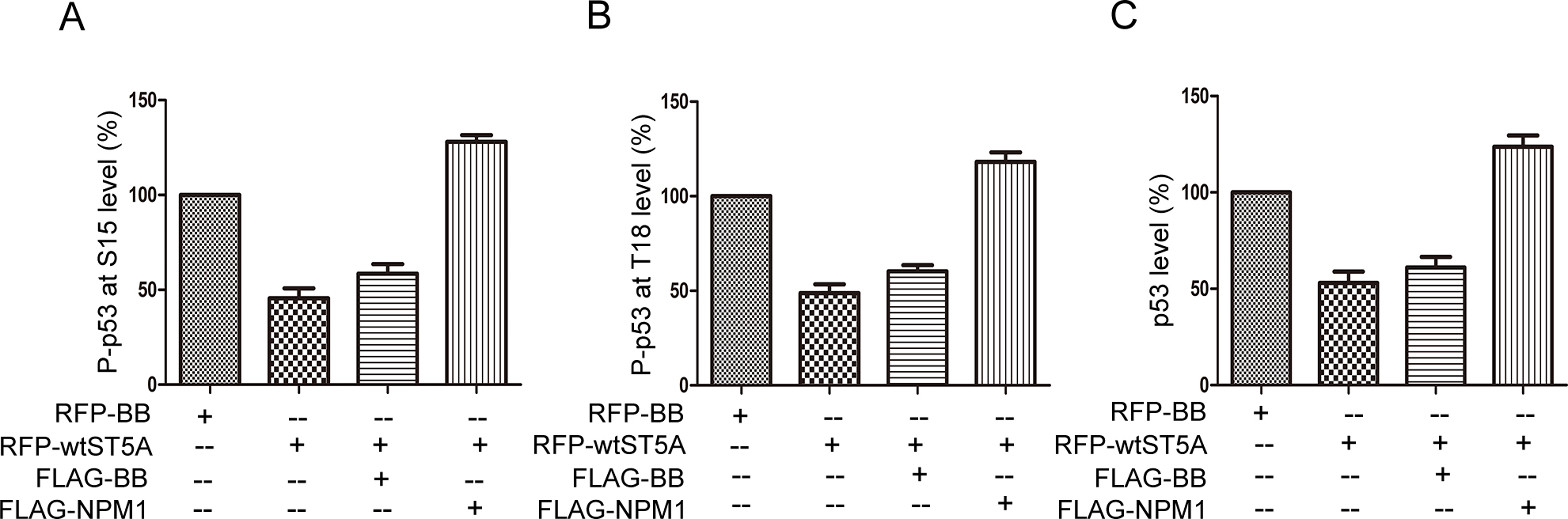

Supplement: Supplementary Figure 10 [file cddis2016430x10.tif]

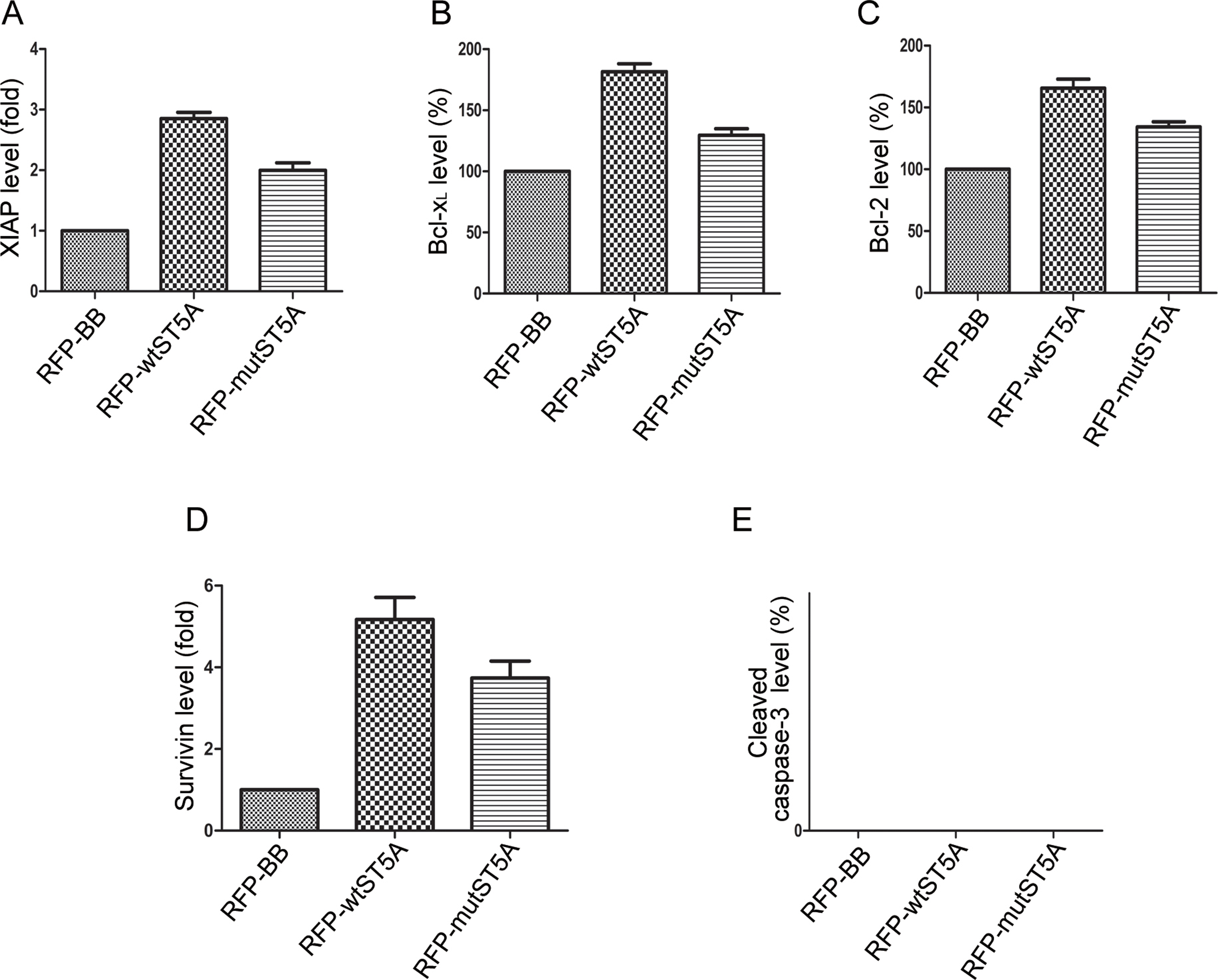

Supplement: Supplementary Figure 11 [file cddis2016430x11.tif]

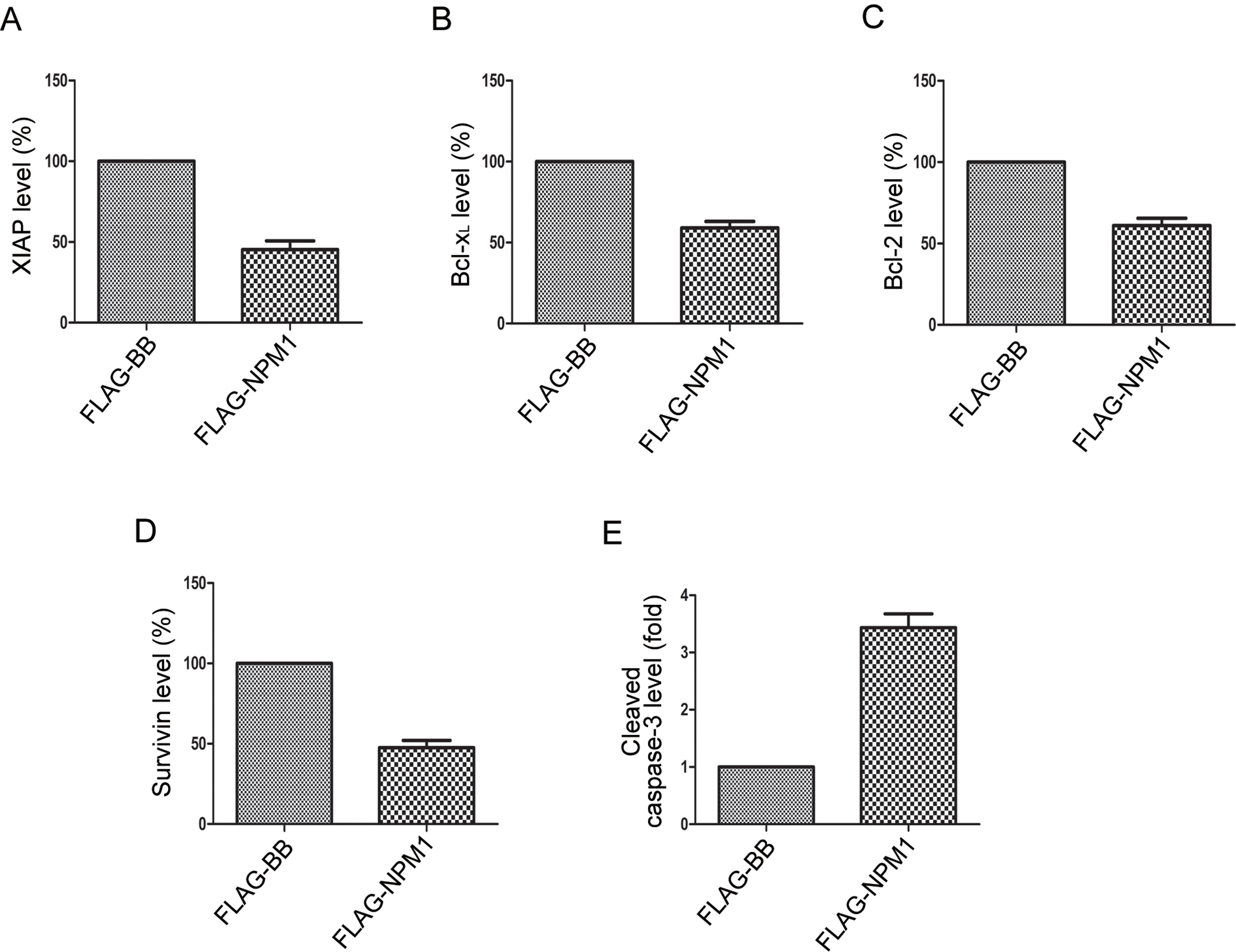

Supplement: Supplementary Figure 12 [file cddis2016430x12.tif]
