# Supplementary material for: Phosphorylated STAT5 regulates p53 expression via BRCA1/BARD1-NPM1 and MDM2
Source: Cell Death Dis. 2016 Dec 22;7(12):e2560–. doi: 10.1038/cddis.2016.430 (PMC5260985; doi:10.1038/cddis.2016.430)
Supplement: Supplementary Table 1 [file cddis2016430x13.docx]

| **Primer Name** | **Sequence** |
| --- | --- |
| RPLPO Forward | 5’- ATGCAGCAGATCCGCATGTCG-3’ |
| RPLPO Reverse | 5’- TGGCACCTTATTGGCCAACAGC-3’ |
| NPM Forward | 5’- TGGAGGAAGATGCAGAGTCA-3’ |
| NPM Reverse | 5’- TTTC TTCA CTGG CGCT TTTT-3’ |

Supplementary Table 1. Primers for the quantitative RT-PCR assays to measure NPM mRNA level.
